# Cycling Promotion and Non-Communicable Disease Prevention: Health Impact Assessment and Economic Evaluation of Cycling to Work or School in Florence

**DOI:** 10.1371/journal.pone.0125491

**Published:** 2015-04-30

**Authors:** Cristina Taddei, Roberto Gnesotto, Silvia Forni, Guglielmo Bonaccorsi, Andrea Vannucci, Giorgio Garofalo

**Affiliations:** 1 Specialization School of Hygiene and Preventive Medicine, University of Florence, Florence, Italy; 2 Quality and Equity Unit, Regional Health Agency of Tuscany, Florence, Italy; 3 Department of Clinical and Experimental Medicine, University of Florence, Florence, Italy; 4 Department of Prevention, Florence Local Health Authority, Florence, Italy; University of New South Wales, AUSTRALIA

## Abstract

**Objective:**

To estimate the effects of cycling promotion on major non-communicable diseases (NCDs) and costs from the public healthcare payer’s perspective.

**Design:**

Health impact assessment and economic evaluation using a dynamic model over a ten-year period and according to two cycling promotion scenarios.

**Setting:**

Cycling to work or school in Florence, Italy.

**Population:**

All individuals aged 15 and older commuting to work or school in Florence.

**Main outcome measures:**

The primary outcome measures were changes in NCD incidence and healthcare direct costs for the Tuscany Regional Health Service (SST) due to increased cycling. The secondary outcome was change in road traffic accidents.

**Results:**

Increasing cycling modal share in Florence from 7.5% to about 17% (Scenario 1) or 27% (Scenario 2) could decrease the incidence of type 2 diabetes by 1.2% or 2.5%, and the incidence of acute myocardial infarction (AMI) and stroke by 0.6% or 1.2%. Within 10 years, the number of cases that can be prevented is 280 or 549 for type 2 diabetes, 51 or 100 for AMI, and 51 or 99 for stroke in Scenario 1 or Scenario 2, respectively. Average annual discounted savings for the SST are estimated to amount to €400,804 or €771,201 in Scenario 1 or Scenario 2, respectively. In Florence, due to the high use of vulnerable motorized vehicles (such as scooters, mopeds, and motorcycles), road traffic accidents are expected to decline in both our scenarios. Sensitivity analyses showed that health benefits and savings for the SST are substantial, the most sensitive parameters being the relative risk estimates of NCDs and active commuting.

**Conclusions:**

Effective policies and programs to promote a modal shift towards cycling among students and workers in Florence will contribute to reducing the NCD burden and helping long-term economic sustainability of the SST.

## Introduction

Physical inactivity has been recognised as one of the leading risk factors for global mortality and non-communicable disease (NCD) burden [1]. Increasing population levels of physical activity is a public health priority. Regular physical activity of moderate intensity is associated with a substantial lower risk of several chronic diseases (e.g., coronary heart disease, stroke, hypertension, type 2 diabetes, colon and breast cancer, depression and cognitive decline), as well as of all-cause mortality [2,3]. Despite these benefits, about a third of the world’s population is not enough physically active. On May 2013 the WHO member states agreed on a 10% reduction in physical inactivity by 2025 as one of the global NCD targets [4], stressing more and more frequently the need to design and implement population-based multi-sectoral policies to address this issue.

There are many good reasons to promote cycling in urban settings, e.g., a better quality of life with less noise, air pollution, and traffic congestion; environmental sustainability, e.g., less fuel consumption; and population health improvement. Indeed, cycling to work or school often does not require extra time and is an easy and convenient way to integrate physical activity into daily life [5,6]. In fact in most cases, it is possible to achieve the recommended level of physical activity simply by commuting by bike. It is cheap; almost everyone can afford it; and leads to possible savings, e.g., fuel and parking costs [6]. Thereby cycling promotion could help to reduce social inequalities in physical activity and health [7,8].

Recent studies have suggested that a modal shift towards active transport, i.e., walking and cycling, in urban areas is feasible [9–11], can improve both population health and the environment [12–20], reduces fossil fuels consumption and may be cost-effective [21–24]. Several cities all over the world have moved in this direction, among them some of the largest metropolitan areas, such as Barcelona, Buenos Aires, Copenhagen, Mexico city, Montreal, New York, Paris, Rio de Janeiro, and Sydney [25–29]. A modal shift towards cycling is indeed possible: cities having implemented cycling promotion policies and infrastructures experienced an increase in cycling levels [10]. For instance, the number of bicycle trips in London increased by 150% between 2000 and 2010 (and by 15% in 2010 alone) [30], and in several Australian cities bicycle commuting increased by 28.9% (by 48.2% in Melbourne) between 2001 and 2006 [31]. However, things can change rapidly depending on social engagement, economic growth and public policies at urban level. For example, in Amsterdam the percentage of people commuting by bike sharply decreased from 75% in 1955 to 25% in 1970. Civil society engaged to promote cycling; a responsive city council adopted effective measures; and cycling levels increased again up to 38% by 2008 [32]. So why not to try in Florence, Italy?

Last year on September 21–29, Tuscany held the 2013 UCI Road Cycling World Championships and 2013 was the Florence “year of cycling”. This international sport event has represented a great opportunity to promote bicycles and make Florence a more bike-friendly city. Our study makes the public health and economic case for cycling promotion, through a health economic assessment providing local decision makers with quantitative evidence of its potential health and economic impact. Specifically we estimated the potential effects of cycling promotion on NCD prevention, namely the reduction in the incidence of type 2 diabetes, acute myocardial infarction (AMI), and stroke, and corresponding savings from the public healthcare payer’s perspective (i.e., Tuscany Regional Health Service).

## Materials and Methods

The health and economic impact of cycling promotion was assessed using a dynamic model over a ten-year period (2013–2022). The study population consisted of all individuals aged 15 and older commuting to work or school/university in Florence (S1 Table). The health economic assessment model used in this study (implemented in Microsoft Office Excel 2003) is available as supplementary file in Supporting Information (S1 File).

### Study population

According to the Italian Behavioural Risk Factor Surveillance System’s data (PASSI Toscana, 2009–2012), in Tuscany, Florence region, 26.9% (95% CI 26.1% to 27.7%) of people aged 18–69 years has a sedentary lifestyle and only 34.7% (95% CI 33.8% to 35.6%) meets the recommended level of physical activity [33]. PASSI’s data also reveal social inequalities in physical activity; the prevalence of a sedentary lifestyle being significantly higher in lower socio-economic groups (S1 Fig).

The 2011 population census carried out by the Italian National Institute of Statistics [34] is the most recent database covering the entire population of Florence. It provides the best data available on mobility in Florence (travel patterns and individuals’ commuting data). It was used to estimate Florence’s modal split, as well as both the proportion and absolute numbers of individuals commuting to work or school by means of transport and travel time. In addition, short-term counts of the number of cyclists in Florence conducted by the Association FirenzeInBici (FIAB) in collaboration with the Municipality of Florence on the first Thursday of October from 2007 to 2010 are consistent with the 2011 population census data. The 2011 population census’ data reveal that in Florence the majority of individuals going to work or school use a car (36.3%) or a motorcycle, moped or scooter (18.9%), and only 7.5% of regular commuters use a bicycle (S2 Fig), cycling an average of 33.1 minutes per person per day. This is true even for short distances, e.g., for transfers to work or school of up to 30 minutes 35.4% of people use a car, 21.4% a motorcycle, moped or scooter, and only 8.5% a bicycle [34].

### Cycling promotion scenarios

Florence could be a “bicycle” city: it’s mostly flat with the exception of hills at the outskirts of the city, with good weather most part of the year, an average annual temperature of 14.9°, min. 9.3° max 20.5°; 88.5 days of rain, and a mean annual rainfall equal to 867.0 mm (Consorzio LaMMA, personal communication). Furthermore, Florence is not too large: almost 50% of commuting journeys to work or school take less than 15 minutes, and 36.5% are between 16 and 30 minutes [34]. Overall, more than 8 trips out of 10 of all commuting to work or school could be made using a bicycle.

In order to estimate the potential health and economic impact of cycling promotion, we modelled two scenario analyses. In the less ambitious Scenario 1, we made the assumption that people using a car or a motorcycle, moped or scooter, going to work/school would switch to a bicycle by a percentage of 25% if the transfer is less than 15 minutes, and by 15% if the transfer is between 16 and 30 minutes. In Scenario 2, we made a more optimistic assumption: people would switch to a bicycle by a percentage of 50% and 30%, if the transfer is less than 15 minutes or between 16–30 minutes, respectively. The time needed to reach the targets being 1 and 3 years in Scenario 1 and 2, respectively.

### Health economic assessment model

#### NCD prevention

Consistent with methodology used by WHO/Europe in the health economic assessment tool (HEAT) for cycling [35], to model the health benefits of physical activity due to regular cycling we used existing evidence of active commuting (walking and cycling) on NCD prevention. As a consequence, we limited our health economic assessment to three health outcomes for which there is evidence of risk reduction due to active commuting: type 2 diabetes, AMI, and stroke. In most previous studies [12,15,18–20,23] the health benefits of cycling were based on the literature on physical activity in general, requiring reliance in several assumptions such as age- and sex- distribution of people cycling and their previous level of physical activity, about how cycling might influence total physical activity, as well as on potential physical activity substitution [35]. Using hazard ratios of active commuting adjusted for other types of physical activity, as in the HEAT tool, allowed us not to make these assumptions.

A first assumption is that the effect of regularly cycling applies only to incident cases of AMI, stroke, and type 2 diabetes’ prevention, with no effect on prevalent cases as well as on other chronic diseases. To estimate the number of incident cases prevented in our two scenarios, we used the hazard ratios (HR) among cyclist compared to non-cyclist commuters for each health outcome. Cyclist commuters’ HRs were taken from prospective studies based specifically on cycling or active commuting and were adjusted for other types of physical activity as well as for main confounding factors (Tables 1 and 2). These data are consistent with results of systematic reviews and meta-analysis considering both overall and leisure-time physical activity [3,40].

**Table 1:** Cycling and NCD prevention data.

	Age- and sex-standardised incidence rate per 100,000	HR among regular commuter cyclists	Time needed for health benefits to build up
**Type 2 diabetes**	760; Bonora et al. 2004 [36]	0.64 (95% CI: 0.45–0.92); Hu et al. 2003 [38]	5 years; WHO/Europe HEAT for cycling [35]
**Acute Myocardial Infarction**	276.3; Regional Health Agency of Tuscany, 2006–2008 [37]	0.82 (95% CI: 0.71–0.95); Hoevenaar-Bloom et al. 2011 [39]	5 years; WHO/Europe HEAT for cycling [35]
**Stroke**	275.5; Regional Health Agency of Tuscany, 2006–2008 [37]	0.82 (95% CI: 0.71–0.95); Hoevenaar-Bloom et al. 2011 [39]	5 years; WHO/Europe HEAT for cycling [35]

**Table 2:** Literature data: Active commuting and NCD prevention.

Active commuting and NCD prevention	Study design & Methods	Study population	Year data were collected & Setting	Results	Reference
**Type 2 diabetes**	Prospective cohort study; To assess the association between specific types of physical activity (occupational, commuting, leisure-time) and type 2 diabetes incidence.	6,898 men; 7,392 women	1982, 1987, 1992	Adjusted Hazard Ratio of type 2 diabetes incidence for walking or cycling to work:	Hu et al. 2003 [38]
	Random sex-age stratified population sample.	Aged 35–64; 48% male	Finland	1–29 min: 0.96 (0.74–1.25)	
	Self-administered questionnaire: medical history, socioeconomic factors, physical activity (occupational, commuting, leisure-time), and smoking; Physical examination: BMI, systolic blood pressure.		(Eastern and southwestern regions, Helsinki capital area)	≥ 30 min: 0.64 (0.45–0.92)	
	Baseline surveys with cohorts in 1982, 1987, 1992, participation rate 74–88%; Mean follow-up period 12 years.			Adjustment: age, sex, study year, other types of physical activity (both occupational and leisure-time), education, systolic blood pressure, smoking, BMI.	
**Acute Myocardial Infarction and Stroke**	Prospective cohort study; To assess the association between specific types of physical activity (walking, gardening, cycling, and sports) and cardiovascular disease incidence (myocardial infarction, angina pectoris, and stroke).	7,451 men; 8,991 women	1994–1997	Adjusted Hazard Ratio of CVD incidence for cycling:	Hoevenaar-Bloom et al. 2011 [39]
	Random sex-age stratified population sample.	Aged 20–65; 45% male	The Netherlands	0.82 (0.71–0.95)	
	Self-reported physical activity (EPIC/MORGEN questionnaire). Survey’s questionnaire: physical activity (leisure time, commuting, sports, and occupational), educational level, smoking, alcohol consumption, CVD risk factor medication; Physical examination: BMI, cholesterol (total and HDL), systolic blood pressure.		(Doetinchem, Maastricht, and Amsterdam)	No evidence of a dose-response relationship.	
	Baseline surveys from 1994 to 1997, average response rate 45%; Mean follow-up period 9.8 years.			Adjustment: age, sex, other types of physical activity (both occupational and leisure-time), smoking, alcohol consumption, education.	

Studies show that there is a substantial lower risk of AMI, stroke, and type 2 diabetes for cycling about 20–30 minutes per day, yet it doesn't seem to be clear evidence of a dose-response in commuting by bicycle and NCD prevention. Due to the fact that in Florence regular commuter cyclists cycle an average of about 30 minutes per person per day and this is the case even for the two scenario analyses, we did not take this aspect into account. Therefore, to calculate the potential impact of cycling promotion on NCD prevention we directly used the adjusted HR estimates of active commuting [38,39]. However, we made a conservative assumption that only 75% of regular commuter cyclists will enjoy better health due to regular cycling. As in the WHO/Europe HEAT for cycling, we assumed that time needed to achieve the full effect on NCD prevention would be 5 years, with an increment of 20% in benefits each year [35].

Age- and sex-standardized incidence rates for each health outcome were obtained from epidemiological studies [36] and regional disease registries (Regional Acute Myocardial Infarction and Stroke Registries of Tuscany) [37] (Table 1). Natural history of incident type 2 diabetes’ cases over a 10-year period was modelled using literature data and public records for Tuscany population. In particular, all-cause mortality in individuals with type 2 diabetes was calculated applying age- and sex-specific relative risks of death from any cause for persons with diabetes compared to their non-diabetic peers in the European population [41] to the corresponding age- and sex-specific all-cause mortality rates in Tuscany residents [42]. Short- and long-term prognoses after a first AMI and stroke event was modelled using results of a retrospective cohort study. Six cohorts of Tuscany residents aged 20–74 years with a first AMI or stroke event in 2001 (AMI n = 4,258; stroke n = 3,515), in 2005 (AMI n = 4,414; stroke n = 3,324), and in 2008 (AMI n = 4,101; stroke n = 3,027) were identified in the Tuscany Acute Myocardial Infarction and Stroke Registries, respectively. These disease registries, through record linkage between Regional Hospital discharge data and the Tuscany Regional Mortality Registry, allow to ascertain both hospitalized cases and out-of-hospital deaths for AMI and stroke among Tuscany residents. First AMI or stroke event was defined as hospital discharge with a principal diagnosis of AMI (code 410*, ICD9-CM) or stroke (code 430*, 431*, 432*, 434*, 436*, ICD9-CM) or out-of-hospital death with cause of death AMI (code 410*, ICD9-CM) or stroke (code 430*, 431*, 432*, 434*, 436*, ICD9-CM). Individuals with an AMI or stroke hospital discharge in the 5 years prior to the index event were excluded. Age was computed at the index event. All these 6 cohorts were followed up through record linkage of health-related administrative databases (Tuscany AMI and Stroke Registries, Tuscany Regional Mortality Registry, Regional Hospital discharge records, Drug dispensing records, Disease-specific exemptions from co-payment to healthcare records) up to December 31, 2011. A follow-up of about 10.5, 6.5, and 3.5 years respectively for 2001, 2005, 2008 cohorts has been performed for all-cause mortality, and in the case of first AMI cohorts for congestive heart failure incidence too.

Both the Tuscany disease registries and the health-related administrative databases’ use for AMI, stroke, and heart failure cases’ ascertainment have been previously validated [43,44], and are being regularly used for NCD surveillance by the Regional Health Agency of Tuscany. The advantage of using results of a retrospective cohort study compared to literature data is the possibility to specifically select Tuscany residents aged 20–74 years (the age group of our health economic assessment study, as result of cycling from age 15 to about 69 years), and to ensure a sufficient long follow-up to model the natural history of individuals with a first AMI or stroke event over a ten-year period.

Finally, AMI, stroke, type 2 diabetes, and heart failure’s direct costs from the public healthcare payer’s perspective were drawn from literature data, by searching PubMed and grey literature between June 2012 and April 2013 (Table 3). Since in a given year deaths may occur at any time, in the model it was assumed that deaths occurred on average in the middle of the year, so that people who died in a given year contributed only 6 months to corresponding healthcare direct costs. All costs were adjusted for inflation (average annual inflation rate 2.2%, Italy 2002–2012) [50] and are reported in Euros (€), 2013 value. Over the 10-year period modelled (2013–2022), a discount rate of 5% per year was applied to future savings [35].

**Table 3:** Literature data: Direct costs from the public healthcare payer’s perspective.

Direct costs from the public healthcare payer’s perspective	Description of the study	Study population	Year data were collected & Setting	Average annual healthcare direct costs per patient:	Reference
**Type 2 diabetes**	To estimate the prevalence and direct costs from the public healthcare payer’s perspective of pharmacologically-treated diabetes in Italy; 10-year longitudinal analysis of prevalence, incidence and direct costs (drug use, inpatient and outpatient activities) for the National Health Service (NHS) of pharmacologically-treated diabetes in 22 Italian Local Health Districts (ARNO observatory, population-oriented database).	311,979 individuals	2006; Italy (22 Local Health Districts)	€2,589 (95% CI, 2,584–2,594)	Marchesini et al. 2011 [45]
**Acute Myocardial Infarction**	To estimate economic burden of hospitalized events of Acute Myocardial Infarction; Study population: all subjects admitted because of first acute myocardial infarction in Lombardy Region in 2003, followed-up until December 31, 2005; Direct costs from the Italian National Health Service (NHS) perspective (drug use, inpatient and outpatient activities).	12,049 individuals	2003; Italy (Lombardy Region)	1^st^ year: €9,136 (SE 123.48); (Hospitalization & acute phase: €6,022)	Mantovani et al. 2011 [46]
				2^nd^ and 3^rd^ year: €2,100 (SE 57.94)	
	Cost-effectiveness analysis of preventive treatment with ramipril in patient at high risk of cardiovascular events; Direct costs from the Italian National Health Service (NHS) perspective (drug use, inpatient and outpatient activities); Resources involved in each event/activity were estimated using the modified Delphi technique with a panel of six clinicians.	1,000 individuals on ramipril; 1,000 individuals on placebo (based on the HOPE trial, 9,297 individuals)	2004; Italy	1^st^ year: €7,504; (Hospitalization: €4,093)	Capri & Perlini 2005 [47]
				subsequent years after the 1^st^ one: €1,795	
**Stroke**	To estimate stroke’s direct costs and productivity losses in Italy from a societal perspective; Prospective incidence-based observational multicentre cost of illness study (EcLIPSE study); Study population: patients admitted because of acute first-ever stroke in 11 Italian hospitals; Costs and outcomes at patients’ enrolment, and at 3, 6 and 12 months after discharge, using a bottom-up approach; Hospital selection: to represent current geographical distribution in Italy and difference in structure and organization (level of care intensity and specificity)- 3 Stroke Units, 4 neurology wards, 4 medicine wards.	449 individuals	2005; Italy (11 Hospitals; 3 Stroke Units, 4 neurology wards, 4 medicine wards)	1^st^ year: €7,611; (Hospitalization & acute phase: €3,252, First 6 months: €6,111)	Gerzeli et al. 2005 [48]
	Cost-effectiveness analysis of preventive treatment with ramipril in patient at high risk of cardiovascular events; Direct costs from the Italian National Health Service (NHS) perspective (drug use, inpatient and outpatient activities); Resources involved in each event/activity were estimated using the modified Delphi technique with a panel of six clinicians.	1,000 individuals on ramipril; 1,000 individuals on placebo (based on the HOPE trial, 9,297 individuals)	2004; Italy	1^st^ year: €17,318 (Hospitalization: €2,751)	Capri & Perlini 2005 [47]
				subsequent years after the 1^st^ one: €1,233	
**Heart failure**	To assess heart failure prevalence, hospitalization rate, adherence to guidelines and social costs; Analysis of heart failure social costs using a retrospective “bottom-up” approach; Healthcare direct costs: drug use, inpatient and outpatient activities.	116 individuals	2001; Italy (Veneto Region)	€3,042	Valle et al. 2006 [49]

**Fig 1:**
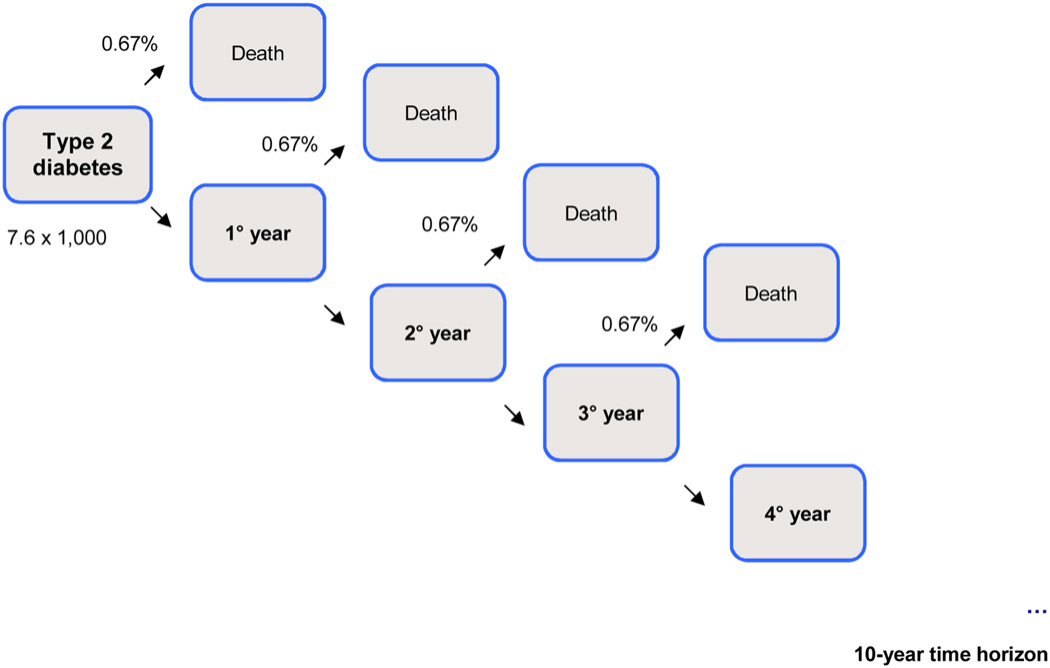
Schematic representation of first 4 years’ model: type 2 diabetes.

**Fig 2:**
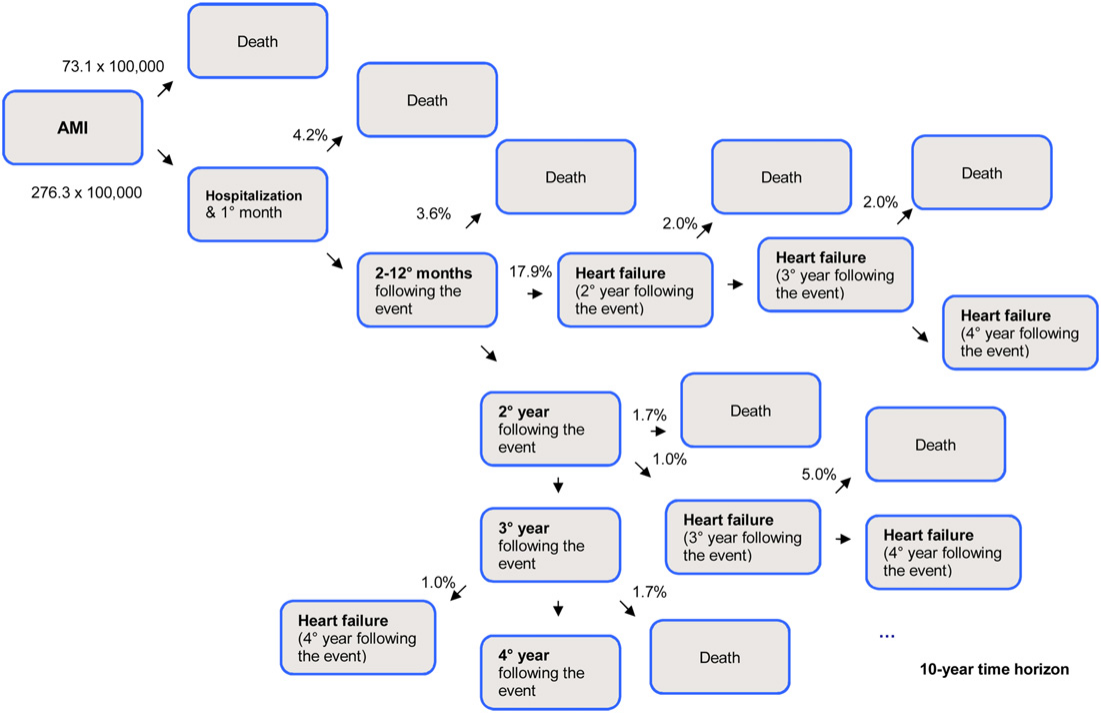
Schematic representation of first 4 years’ model: acute myocardial infarction.

**Fig 3:**
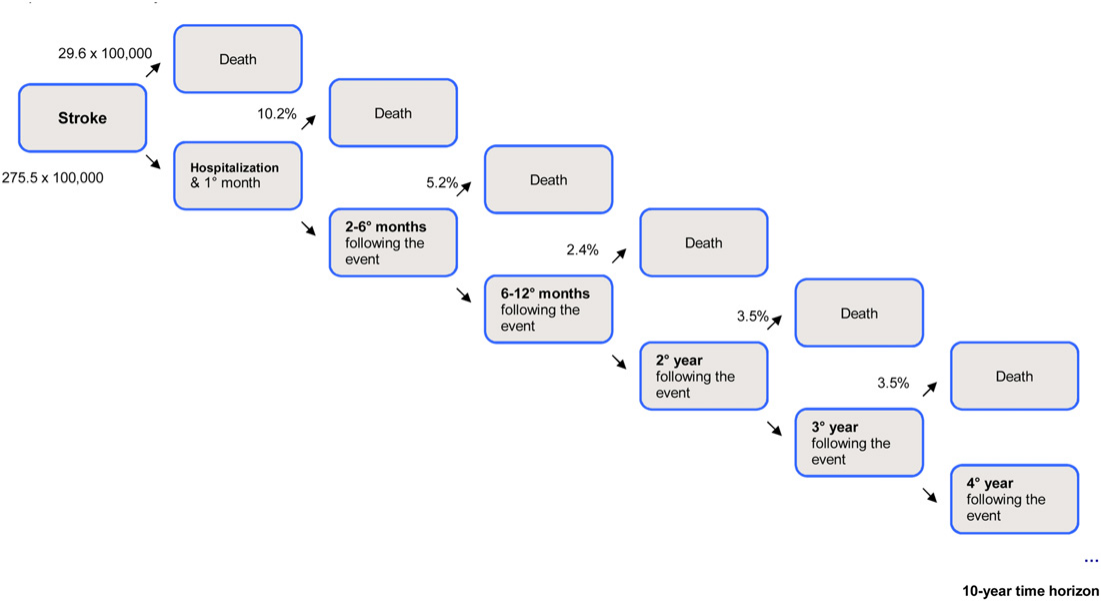
Schematic representation of first 4 years’ model: stroke.

Model parameters and input data are summarized in Table 4, and an overview of the first 4 years of the health economic assessment model is schematically outlined in Figs 1–3.

**Table 4:** Health economic assessment model data.

Selected health outcomes	Natural history of selected health outcomes			Average annual cost per patient (€, 2013 value)		
	1^st^ year	2–5° year	6–10° year	1^st^ year	2^nd^ year	3–10° year
**Type 2 diabetes**	M: 0.67%	M: 0.67%	M: 0.67%	€3,015; Marchesini et al. 2011 [45]	€3,015; Marchesini et al. 2011 [45]	€3,015; Marchesini et al. 2011 [45]
**AMI**	28-day M: 4.2%	M: 1.7%	M: 2.3%	Hospitalization & 1° month: €6,290; Mantovani et al. 2011 [46]	€2,180; Capri & Perlini 2005 [47]	€2,180; Capri & Perlini 2005 [47]
	2–12° month M: 3.6%			2–12° months: €9,540; Mantovani et al. 2011 [46]		
	HF: 17.9%	HF: 1.0%	HF: 1.0%			
**Stroke**	28-day M: 10.2%	M: 3.5%	M: 3.5%	Hospitalization & 1° month: €4,220; Gerzeli et al. 2005 [48]	€2,470; Gerzeli et al. 2005 [48]	€1,500; Capri & Perlini 2005 [47]
	2–6° month M: 5.2%			2–6° months: €3,715; Gerzeli et al. 2005 [48]		
	6–12° month M: 2.4%			6–12° months: €1,945; Gerzeli et al. 2005 [48]		
**Heart failure in the 1** ^**st**^ **year following AMI**	M: 2.0%	M: 2.0%	M: 2.4%	€4,035; Valle et al. 2006 [49]	€4,035; Valle et al. 2006 [49]	€4,035; Valle et al. 2006 [49]
**Heart failure in the 2–10° year following AMI**	M: 5.0%	M: 3.3%	M: 3.3%	€4,035; Valle et al. 2006 [49]	€4,035; Valle et al. 2006 [49]	€4,035; Valle et al. 2006 [49]

M: all-cause mortality

HF: Heart failure incidence following AMI

#### Road traffic accidents

The potential impact of cycling promotion on road traffic accidents and fatalities was assessed with a risk- and travel time-based model. Florence road traffic accidents and fatalities by mode of transport were obtained from police-reported data and refer to Florence municipal area over the period 2008–2010 [51]. Florence mobility statistics and travel time by means of transportation were drawn by 2011 population census [34].

Road traffic accidents and deaths’ risk was calculated as the number of both road traffic accidents and deaths per billion passenger minutes travelled by bicycle, car, and motorcycle, moped or scooter, respectively, assuming 232 working/education days (Table 5). First, these data suggest that in Florence there are about 52% and 69% less road traffic accidents per minute travelled by bicycle than by car or motorcycle, moped or scooter, respectively. Second, cyclists are about 1.4 times more likely than car drivers to have a fatal road traffic accident, whereas there are about 84% less road traffic deaths per minute travelled by bicycle than by motorcycle, moped or scooter.

**Table 5:** Exposure to road traffic accidents and deaths by means of transportation (assuming 232 working/education days).

	Rate per billion passenger minutes travelled			Risk Ratio	
	Bicycle	Car	Motorcycle, moped or scooter	bicycle/car	bicycle/ motorcycle, moped or scooter
**Road traffic accidents**	3004.9	6219.3	9730.8	0.48	0.31
**Road traffic deaths**	6.4	4.6	39.1	1.39	0.16

The change in absolute numbers of road traffic accidents and deaths was estimated based on the amount of minutes shifted from travel by car or motorcycle/moped/scooter to bicycle, according to the two cycling promotion scenarios (Table 6) and assuming the same travelled time for each means of transportation, similarly as done by previous studies [13,14]. In Florence, due to the high percentage of regular motorcycle, moped or scooter commuters (21.4% for transfers of up to 30 minutes), in both our scenario analyses we expect a decrease both in road traffic accidents and deaths. Indeed, road traffic accidents and deaths would decline respectively about 6.9% and 6.2% in Scenario 1, and about 13.9% and 12.5% in Scenario 2. This reduction may be even larger than we estimated due to the “safety-in-numbers” effect [52,53].

**Table 6:** Road traffic accidents and deaths by means of transportation, 2008–2010 Municipality of Florence data and cycling promotion scenario analysis.

	Road traffic accidents			Road traffic deaths		
	2008–2010 data, annual average [51]	Scenario 1	Scenario 2	2008–2010 data, annual average [51]	Scenario 1	Scenario 2
**Bicycle**	315	664	1,013	0.7	1.4	2.1
**Car**	4,072	3,618	3,165	3.0	2.7	2.3
**Motorcycle, moped or scooter**	2,652	2,231	1,809	10.7	9.0	7.3
**Other (e.g., walking, public transport)**	537	537	537	6.3	6.3	6.3
**Total**	7,576	7,050	6,524	20.7	19.4	18.1
**Difference (%)**		-526 (-6.9%)	-1,051 (-13.9%)		-1.3 (-6.2%)	-2.6 (-12.5%)

However, due to the huge difference in risks of road traffic accidents and deaths per minute travelled by car compared to motorcycle/moped/scooter, the population impact of a modal shift towards cycling on the number of both road traffic accidents and deaths in Florence will largely depend on which commuters (car versus motorcycle/moped users) will switch to bicycle. For instance, if the modal shift towards cycling is all from motorcycle, moped or scooter users, the estimated reduction in road traffic accidents and deaths would be much larger (respectively, -10.3% and -18.4% in scenario 1; -20.6% and -36.9% in scenario 2). Yet, if it is all from car users we will still be expecting an overall reduction in road traffic accidents (-4.9% and -9.9% in Scenario 1 and Scenario 2, respectively), whereas the number of road traffic fatalities would increase (+1.0% and +2.0% in Scenario 1 and Scenario 2, respectively). In addition to this, the impact of cycling promotion on road traffic accidents and fatalities will depend on factors such as cycling safety policies and infrastructures (i.e., bike lanes, 30 km/h zones), cyclists trajectories and behaviours, as well as on a potential “safety in numbers” effect and on motor vehicle users’ behaviours.

Due to the great uncertainty related to road traffic accidents’ modelling we decided not to include it in the final health economic assessment. Anyhow, several studies that took this aspect into account show that the health benefits of cycling largely outweigh the risks [12–16,18–20,23,24].

### Sensitivity analysis

To test the robustness of our results, we ran several sensitivity analyses using alternative values for model assumptions and key parameters (Table 7).

**Table 7:** Model assumptions, key parameters and sensitivity analysis.

Model assumptions and key parameters	Main analysis	Sensitivity analysis
**Cycling promotion scenarios: percentage of commuters using a car or a motorcycle, moped or scooter, that would switch to a bicycle going to work or school**	Transfers of up to 15 minutes:	Transfers of up to 15 minutes:
	Scenario 1: 25%	Scenario 1: 20–30%
	Scenario 2: 50%	Scenario 2: 40–60%
	Transfers between 16 and 30 minutes:	Transfers between 16 and 30 minutes:
	Scenario 1: 15%	Scenario 1: 10–20%
	Scenario 2: 30%	Scenario 2: 25–35%
**Regular commuter cyclists enjoying better health due to their regular physical activity**	75%	50–100%
**Time needed to reach full level of cycling**	Scenario 1: 1 year	Scenario 1: 0–3 years
	Scenario 2: 3 years	Scenario 2: 1–5 years
**Time needed for health benefits to build up**	5 years	3–8 years
**Yearly discount rate**	5%	3.5%
**Relative risks estimates**	HR	upper and lower bounds of the 95% CI
**Healthcare direct costs**	literature data, adjusted to €2013	-/+ 20%

Cycling promotion scenarios’ assumptions were tested using a worst- and best-case scenarios approach. That is, in Scenario 1 people using a car or a motorcycle going to work or school would switch to a bicycle in a percentage of 20–30% if the transfer is less than 15 minutes and of 10–20% if the transfer is between 16 and 30 minutes. Whereas, in Scenario 2 people would switch to a bicycle in a percentage of 40–60% and 25–35% if the transfer is less than 15 minutes and between 16–30 minutes, respectively. The number of regular commuter cyclists who benefit from regularly exercising was changed by 25% from the assumed 75% (50–100%). The time needed to reach the full level of cycling was changed from 1 year to 0–3 years in Scenario 1, and from 3 years to 1–5 years in Scenario 2. In the main analysis, we assumed a 5 years lag time before the full effect on NCD prevention was achieved. We tested this assumption by using a 3–8 years lag time. In addition, we undertook a sensitivity analysis varying the discount rate from 5% to 3.5% per year. Finally, the uncertainty associated with the relative risk estimates was tested by using the upper and lower bounds of the 95% CI of the HRs. Healthcare direct costs were changed by 20%.

As far as stroke is concerned, a large amount of the financial burden is due to non-healthcare direct costs, such as paid care and home adaptations, which are borne by the patient’s family or social welfare. To evaluate the potential impact of cycling promotion on this aspect, we carried out a sensitivity analysis taking into account total direct costs attributable to stroke.

Lastly, the health economic impact of cycling promotion was estimated by the change in population physical activity levels using potential impact fraction (PIF).

### Software for data analysis

For data analyses we used both STATA 11.0 and Microsoft Office Excel 2003, for the health economic assessment model we used Microsoft Office Excel 2003.

## Results

According to our scenario analyses, the less ambitious cycling promotion strategy (Scenario 1) would result in 17,292 additional individuals regularly cycling an average of 30.8 minutes per person per day (Table 8). Over 10 years, the number of cases that can be prevented by such an increase in cycling is 280 for type 2 diabetes, 51 for AMI, and 51 for stroke, resulting in 5% discounted savings on healthcare direct costs of €4,008,037. This change would also let us prevent 7 cases of heart failures and 42 premature deaths (Table 9). In the optimistic cycling promotion strategy (Scenario 2), new regular commuter cyclists would be 34,583 cycling an average of 30.1 minutes per person per day (Table 8). Over the same ten-year period, 549 cases of type 2 diabetes, 100 cases of AMI and 14 of heart failure, 99 cases of stroke, and 82 premature deaths would be prevented (Table 9). The corresponding savings from the public healthcare payer’s perspective are estimated to amount to €7,712,006 discounted by 5% per year.

**Table 8:** Individuals commuting to work or school in Florence, by mode of transport and travel time, 2011 population census data and cycling promotion scenario analysis (rounded numbers).

	≤ 15 minutes			16–30 minutes			> 30 minutes	Total		
	2011 data	Scenario 1	Scenario 2	2011 data	Scenario 1	Scenario 2	2011 data, Scenario 1, and Scenario 2	2011 data	Scenario 1	Scenario 2
**Walking**	28,278			5,343			722	34,343		
**Bicycle**	7,707	18,792	29,878	5,116	11,322	17,527	795	13,617	30,909	48,200
**Car**	26,397	19,798	13,200	27,008	22,957	18,906	12,465	65,873	55,220	44,571
**Motorbike, moped, scooter**	17,944	13,458	8,973	14,363	12,209	10,054	2,028	34,336	27,695	21,055
**Public transport**	3,651			13,697			14,295	31,643		
**Other**	662			715			303	1,680		
**Total**	84,637			66,242			30,613	181,491		

Scenario 1: Less ambitious cycling promotion target

Scenario 2: Optimistic cycling promotion target

**Table 9:** NCD prevention and healthcare direct costs savings (5% discount rate per year) based on Florence cycling promotion scenarios over a ten-year period (2013–2022).

	Incident cases prevented over a 10-year period (2013–2022)		Premature deaths prevented over a 10-year period (2013–2022)		Maximum decrease in annual incidence*		Potential savings from the public healthcare payer’s perspective, discounted by 5% per year (€, 2013 value)	
	Scenario 1	Scenario 2	Scenario 1	Scenario 2	Scenario 1	Scenario 2	Scenario 1	Scenario 2
**Type 2 diabetes**	280 (211–350)	549 (413–685)	4 (3–5)	8 (6–9)	1.7% (1.3%-2.1%)	3.3% (2.5%-4.1%)	2,714,296 (2,041,530–3,386,910)	5,172,991 (3,890,926–6,454,903)
**AMI**	51 (38–64); HF: 7 (5–9)	100 (75–124); HF: 14 (11–18)	19 (14–24)	37 (28–47)	0.8% (0.6%-1.0%)	1.7% (1.3%-2.1%)	753,697 (566,886–940,466)	1,479,008 (1,112,453–1,845,521)
**Stroke**	51 (38–63)	99 (75–124)	19 (14–24)	37 (28–46)	0.8% (0.6%-1.0%)	1.7% (1.3%-2.1%)	540,044 (406,189–673,869)	1,060,007 (797,296–1,322,687)

Scenario 1: Less ambitious cycling promotion target

Scenario 2: Optimistic cycling promotion target

HF: Heart failure

Worst- and best-case cycling promotion scenarios are given in brackets

* Yearly reduction in incident cases at full effect, 100% of regular commuter cyclists enjoying better health due to their regular physical activity.

As far as NCD prevention is concerned, the estimated impact of cycling promotion in Scenario 1 would be a decrease in the annual incidence of 1.2% for type 2 diabetes, and of 0.6% for AMI and stroke. Whereas if Scenario 2’s increase in cycling could be reached, the expected incidence reduction would be 2.5% for type 2 diabetes, and 1.2% for AMI and stroke.

From an economic perspective, every new regular commuter cyclist would allow the Tuscany Regional Health Service (SST) to save about €230 (discounted by 5% per year, 2013 value). Most of direct healthcare savings would be explained by type 2 diabetes’ prevention, which would allow the SST to save about €2.7 and €5.2 million over the ten-year period (discounted by 5% per year, Scenario 1 and 2 respectively). This is partly explained by approximately three times higher incidence of type 2 diabetes compared to AMI and stroke, and thus to the higher number of cases that could be prevented.

**Fig 4:**
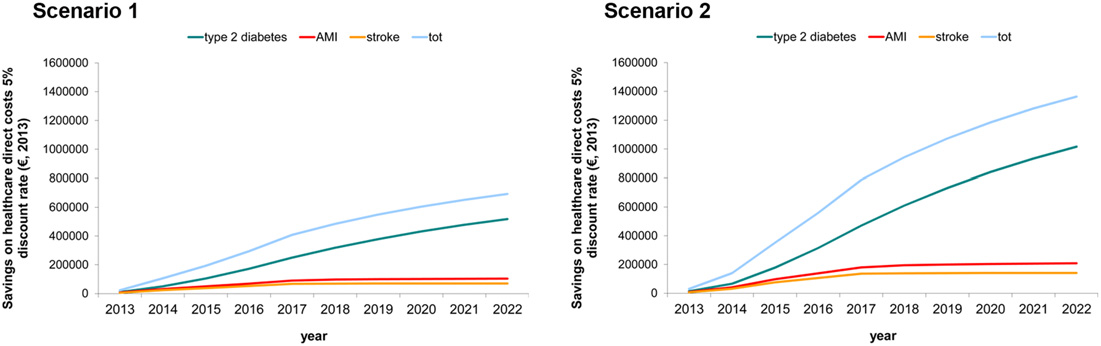
Potential yearly discounted savings on healthcare direct costs by year and health outcome, 5% discount rate (€, 2013). Scenario 1: Less ambitious cycling promotion target; Scenario 2: Optimistic cycling promotion target.

Fig 4 shows potential yearly savings on healthcare direct costs (discounted by 5% per year), expected in the two cycling promotion strategies over the ten-year period 2013–2022. By the first five years, about €1–2 million could be saved (discounted by 5% per year, in Scenario 1 and 2 respectively). In 2022, savings on healthcare direct costs are estimated to be about €0.7–1.4 million (discounted by 5% per year, in Scenario 1 and 2 respectively), with yearly savings further increasing even more in the near future beyond the 10-year period analysed. This is particularly true in the case of type 2 diabetes’ prevention. Indeed, how Fig 4 shows yearly savings are still raising at the end of the 10-year period. By contrast, yearly savings due to AMI and stroke’s prevention seem to reach a plateau by the end of the 10-year period.

**Fig 5:**
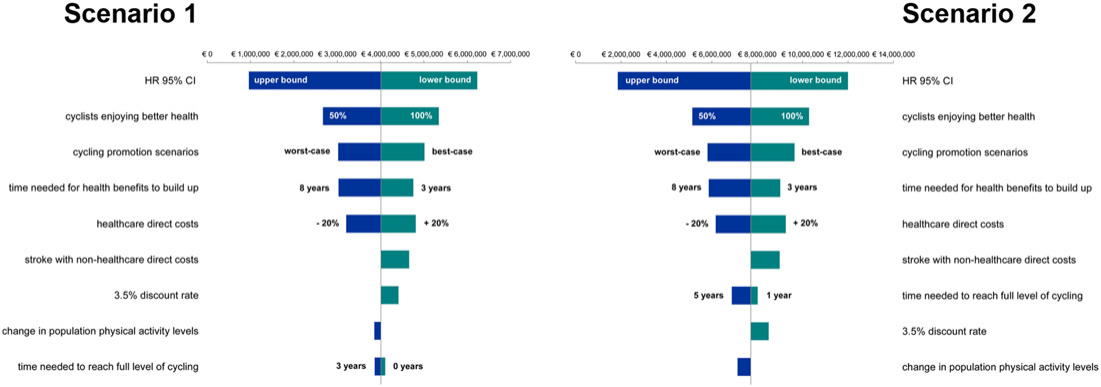
Discounted savings over a 10-year period (2013–2022)—Sensitivity analyses’ results. Scenario 1: Less ambitious cycling promotion target; Scenario 2: Optimistic cycling promotion target.

Sensitivity analyses’ results are shown in Fig 5. Overall, the most sensitive parameter appeared to be HR estimates, with savings from the public healthcare payer’s perspective ranging between about €1–1.9 and €6.2–12.0 million using respectively the upper and lower bounds of the 95% CI (-76% and + 55%; Scenario 1–2, respectively). Changing the percentage of new regular commuter cyclists enjoying better health due to regular exercise to 50% or 100% would impact on healthcare savings by 33%, whereas increasing the lag time before full effect on diseases’ prevention was achieved to 8 years would reduce savings by 24%, with a larger impact on type 2 diabetes related savings (-27%) compared to AMI and stroke (-18%). Taking into account total direct costs of stroke increases stroke related savings by about 120%, confirming the impact of stroke’s financial burden on family and social welfare. Nevertheless, the impact on overall savings is only a 16% increase, well below the estimated impact attributable to other assumptions and parameters’ uncertainty.

Time needed to reach full level of cycling as well as calculating the potential benefits of cycling through the corresponding change in population physical activity levels had a negligible impact on overall savings.

## Discussion

The bicycle is the primary means of transportation for more than 13,000 Florence residents or 7.5% of all commuters. Study results suggest that a modal shift towards bicycle could decrease by 1.2–2.5% the incidence of type 2 diabetes, and by 0.6–1.2% the incidence of AMI and stroke. Average annual savings for the SST, discounted by 5% per year, are estimated to amount to €400,804–771,201, corresponding to about 0.1% (0.06%-0.11%) of average annual total health expenditure in Florence Municipality [54,55]. These savings could be reinvested in health promotion and prevention programs further improving population health, as well as help long-term economic sustainability of the SST.

Several studies evaluated the health impact of cycling [13–15,20,21], active travel [17,19,24], or sustainable mobility promotion [12,16,18], both in terms of mortality [13,14,16,24] and morbidity [12,15,17–20], all of them showing how the health benefits of increased cycling largely outweigh risks.

Furthermore, previous studies have attempted to monetise the value of health benefits due to a modal shift towards sustainable mobility. Lindsay *et al*. [21] estimated changes in air pollution, road safety, and physical activity level, as well as resulting health benefits and monetary costs, due to increasing bicycle commuting instead of light motor vehicle use in New Zealand urban areas. A 5% modal shift in bike favour would reduce transport-related greenhouse emissions by 0.4%. Including only fatalities and using the New Zealand Value of Statistical Life, the health benefits of such a modal shift would amount to net savings of about NZ$200 million (€127.6 million) per year in a population of about 2.7 million people. Grabow *et al*. [22] estimated the health and environmental effects of the elimination of car round trips ≤ 8 km (urban and suburban), in 11 metropolitan areas in the upper midwestern US. The health benefits of reduced air pollution and increased physical activity if 50% of these short trips were made by bicycle would result in net savings of more than US$8 billion (€5.96 billion) per year in a population of 31.3 million. Both these studies resulted in net savings per person much greater than those estimated in our assessment, but they either combine the monetised value of health benefits together with healthcare savings or specifically relate to the monetised value of lives saved using a “value of statistical life” approach. This approach is the one also used by the WHO HEAT for cycling. Using WHO HEAT for cycling, we estimated that the monetised value of lives saved in our two scenario analyses would amount to about €15.4–30.7 million per year, in line with what has been found by Lindsay *et al*. [21].

To our knowledge, only one study has specifically modelled the impact of increasing active travel on costs from the public healthcare payer’s perspective [23]. Jarrett and colleagues [23] estimated that increasing walking and cycling in urban England and Wales, over a 20-year period would result in savings of about UK£17 billion (€21.5 billion) for the National Health Service (NHS) due to the health benefits of increased physical activity on type 2 diabetes, dementia, cerebrovascular disease, breast cancer, colorectal cancer, depression, and ischaemic heart disease’s prevention and after adjustment for an increased risk of road traffic accidents. Compared to Jarrett *et al*. [23] our study specifically assessed the impact of cycling promotion, modelled a much smaller population (a single urban area: Florence Municipality), over a shorter period (10 years), and took into account only three health outcomes—type 2 diabetes, AMI, and stroke. Despite strong evidence of beneficial effects of physical activity on several other diseases, such as breast and colon cancer, depression, and dementia, we have not taken them into account because of lack of active commuting specific evidence. Anyhow, for most of these diseases the lag time between the change in physical exercise and disease prevention may be rather large, e.g., 17 years for breast and colon cancer [23], thus their impact over a ten-year period may be limited and would be seen over a longer run instead, e.g., 20/30 years [23].

Following the methodology proposed in the WHO HEAT for cycling, our model uses HRs based on active commuting adjusted for other types of physical activity as well as for main confounding factors. This allowed us not to make several assumptions (e.g., age- and sex- distribution of people cycling and their previous level of physical activity, potential physical activity substitution). However, this assumption was tested in the sensitivity analysis in which the potential health benefits of cycling were calculated through the corresponding changes in population physical activity levels. Sensitivity analysis’ results were reassuring about the use of active commuting evidence: indeed using either relative risks specific for active travel or based on overall physical activity gave similar results.

There are a number of strengths and limitations to the present study. To the best of our knowledge, it is the first study to specifically assess the impact of cycling promotion on costs from the public healthcare payer’s perspective; to model cycling promotion scenarios in Italy; and it is the first one using specific active commuting evidence on NCD prevention. Discussing this work with local policy makers helped us in constructing our cycling promotion scenarios.

Another strength of the study is the use of high quality data relevant to Florence population. Where possible, it was based on local/national data. In all cases, we used the most reliable data and whenever possible validated them with secondary sources. To analyse the Florence commuting pattern and construct cycling promotion scenarios, we used high quality data covering the entire population at the individual and trip level [34]. Modelling the effects of cycling promotion we drew on large datasets covering multiple years for AMI and stroke, and on literature data for type 2 diabetes as well as for healthcare direct costs. Road traffic accidents and fatalities were modelled using police-reported data [51]. Although police reported data were found to be accurate [56], cyclists minor accidents may be underreported.

Modelling the potential impact of cycling promotion implied data choices and assumptions, which were tested as far as possible through sensitivity analyses. HRs’ estimates turned out to be the most sensitive parameter. Anyhow, even using the upper bound of HRs’ estimates, sensitivity analysis show that increasing cycling levels in Florence would result in substantial health benefits and savings for the SST.

One study limitation is that we were able to include in our model only health outcomes for which there was specific active commuting evidence. As a consequence the potential impact of increased cycling levels on other chronic diseases (e.g., breast and colon cancer, depression, and dementia) as well as on overweight and obesity was not assessed, thus the total benefits may be greater than shown by our results. A further limitation in our modelling is that we have not stratified by age group and sex, i.e., assuming that the potential for a modal shift towards cycling would be the same in all commuters independently of age and sex. Thus, we can only present aggregated results based on a modal shift across the Florence population aged 15 and older. Moreover, empirical evidence on the dose-response relation between active commuting and NCD risk is not yet available, thus in our modelling we used single HR estimates not taking dose-response into account. However, in Florence individuals commuting by bike cycle an average of about 30 minutes per day, which is more than sufficient to reach the recommended level of physical activity [2]. Potential physical activity substitution or compensation mechanisms were not taken into account either. Anyhow, we made the conservative assumption that only 75% of new commuter cyclists will enjoy better health due to regular cycling. Overall, in our modelling we chose a conservative approach, such that the health and economic benefits are likely to be greater.

The feasibility of our two cycling promotion scenarios can be questioned. They imply that in Florence, among all regular commuters about 17.0% or 26.6% would use a bicycle going to work or school, in Scenario 1 or Scenario 2 respectively. It represents an ambitious yet not unrealistic target: in several Italian cities the percentage of urban trips done by bicycle is well over 20%, i.e., Ferrara 27%, Pesaro 28%, and Bolzano 29% [57]. Moreover, our ambitious cycling promotion scenario is still far away from a city like Amsterdam were cycling accounts for about 38% of all urban trips [32]. In addition to this, according to a 2012 Tuscany Region survey on bicycle use [58], in Florence metropolitan area 34.1% of people never using a bicycle is not using it because of traffic and infrastructure’s reasons (traffic accounted for 14.2%, difficult/uncomfortable itineraries for 7.5%, lack of bike lanes for 4.7%). Furthermore, among people not using a bicycle in Tuscany urban areas 33.9% would be willing to use it, providing bike lanes and paths (18.8%) or bike-favour areas (6.2%), less traffic congestion (10.3%), better road surface (5.8%), safe bike parking (3.3%) and parking facilities (1.7%), bike sharing programme (3.2%), cycling subsidies (1.6%), and allowing bicycles on public transport (1.4%).

Recent reviews reported that cycling promotion programs and policies can result in a modal shift towards bicycle [9,11]. Furthermore, there are several examples all over the world revealing how bicycle friendly politics can increase cycling levels in a population. In Copenhagen, Denmark, the percentage of people cycling to work or school increased from 30% in 1998 to 36% in 2012 [27]. In New York City, all-year cycling volumes in 2012 were 58% greater than in 2008 [59]. Overall, commuter cycling in New York City is estimated to be almost four times greater in 2011 than in 2000 [60].

From a cost-benefit perspective, assuming an average bike paths’ cost of €79,600 per km (min-max: €22,521–152,508) [61] healthcare savings freed up due to NCD prevention alone could cover the cost of adding about 50 km of bike paths, that is almost doubling the Florence current cycle path network (89 km) [62], in 7–10 years. This would allow Florence reaching a bike path density of about 135.7 km per 100 km^2^, near to the value that could be found in cities such as Padova, Modena, Bolzano where a greater percentage of people use a bike to commute [62].

Due to widespread reliance on vulnerable modes of motorized transport (such as scooters, mopeds, and motorcycles), our road traffic accidents’ analysis shows that increasing the number of cyclist commuters in Florence could lead to an unexpected positive result: a reduction of both road traffic accidents and fatalities. A similar result has also been found by Woodcock and colleagues [19]. Their study suggests that the number of road traffic injuries would not increase with a modal shift towards active travel as long as there are sufficient reductions in motor vehicle use and lower motor vehicle speeds. Finally, in Florence the population impact of cycling promotion on road traffic accidents will largely depend on which commuters (car versus motorcycle/moped users) will switch to bicycle as well as on a number of different factors (e.g., cycling safety policies and infrastructures), or even on a potential “safety in numbers” effect. Yet increasing the number of cyclists and not the number of cyclists’ accidents, injuries and fatalities is possible. Evidence suggests that providing a comprehensive and well-maintained bike network, suitable transport planning and safety measures may encourage people to cycle as well as improve cyclists’ safety [35]. For instance, in London, cycling increased by 150% between 2000 and 2010, whereas the number of cyclists killed or seriously injured in 2010 fell by 18% compared to the 1994/98 baseline (with an overall 9% decrease in cycling casualties, a 32% decrease in cycling fatalities, a 17% decrease in serious injuries, and a 8% decrease in slight injuries) [30]. In Copenhagen between 1996 and 2012 the percentage of bicycle traffic increased by 20% and the number of injured persons per bike kilometre decreased by almost 60% [27]. Since 2000, the number of trips by bike in New York City has nearly tripled while the average risk of serious injury experienced by cyclists has fallen by 72% [63].

Due to the great uncertainty related to road traffic accidents’ modelling we decided not to include it in the final health economic assessment. Air and noise pollution were not modelled either. However, how several studies have already shown the overall benefits of cycling largely outweigh risks (i.e., road traffic accidents and air pollution). Furthermore, a modal shift towards bicycle along with a reduction in motor vehicles’ use may increase road traffic safety for the general population as well as reduce urban traffic noise and pollution. Reductions in greenhouse-gas emissions will not only be relevant for reducing climate change and improving air quality and environment, they will also add further population health benefits.

In Tuscany, the prevalence of a sedentary lifestyle is significantly higher in lower socio-economic groups (PASSI 2009–2012 [33], S1 Fig) and some evidence suggests that cycling promotion could help closing this gap [7,8]. Assessing the impact of cycling promotion on health inequalities was beyond the scope of our study, but it undoubtedly deserves further research and attention.

As for our modelling approach, we chose the public healthcare payer’s perspective, thus the wide economic or social effects of cycling promotion were not considered. Furthermore, to make an economic case for cycling promotion we modelled the likely health and economic impact in the short to medium term (10-year time horizon). Due to the lag period for health benefits to build up study results show that savings to the SST are still rising at the end of the 10-year study period suggesting that further savings could be expected in the long run.

Finally, it may be argued that preventing NCDs and premature deaths today may lead to a higher disease burden in an older population in the near future. That is, today’s savings will be offset by tomorrow’s healthcare costs. Yet there is strong evidence that lifestyles’ interventions are associated with healthy ageing: promoting healthy lifestyles today will lead to a compression in morbidity with a potential for further healthcare savings in future [64–66].

## Conclusions

The bicycle is a healthy and green means of urban transportation, an alternative to motor vehicles, and a promising way to increase physical activity in urban communities.

Study results suggest that cycling promotion could provide a substantial reduction in NCD burden, as well as savings for the SST. The population health gain due to increased physical activity would be just one, yet significant, of cycling promotion benefits.

Unlike motor vehicles the bicycle is clean: does not produce air and noise pollution. The bicycle is space- and energy-efficient: it reduces waste, dependence on fossil fuels and traffic congestion, improving both environment and quality of life in urban areas. Furthermore, cycling promotion could even unlock new opportunities: WHO Europe concluded that more than 70,000 jobs could be created if selected cities increased their cycling levels as done in Copenhagen [67].

Last year, during the 2013 UCI Road Cycling World Championships Florence discovered that “another” mobility is not only possible, but also worthy. Now it's time to make that awareness a reality of everyday life, through public policies and social engagement. Indeed, increasing the number of individuals cycling to work or school in Florence not only is realistically feasible but also a win-win approach: it improves population health and environment, and is cost-effective.

## Supporting Information

S1 CHEERS Checklist CHEERS Checklist. (DOC)

S1 Fig Prevalence of a sedentary lifestyle by socio-economic level in Tuscany. Italian Behavioural Risk Factor Surveillance System’s data (PASSI Toscana, 2009–2012) [33]. Overall, among people aged 18–69 years with an Italian citizenship (n = 13,262) the prevalence of a sedentary lifestyle is 26.5% (95% CI 25.7% to 27.4%).
(TIF)

S2 Fig Commuting to work or to school in Florence by means of transport. Italian National Institute of Statistics (ISTAT), 2011 population census [34].
(TIF)

S1 File Health economic assessment model. (XLS)

S2 File Retrospective cohort study, AMI data. (XLS)

S3 File Retrospective cohort study, stroke data. (XLS)

S1 Table Florence population by sex and age-group, 2011 population census data. Italian National Institute of Statistics (ISTAT), 2011 population census [34].
(DOCX)
