# Supplementary figures and images for: Cycling Promotion and Non-Communicable Disease Prevention: Health Impact Assessment and Economic Evaluation of Cycling to Work or School in Florence
Source: PLoS One. 2015 Apr 30;10(4):e0125491. doi: 10.1371/journal.pone.0125491 (PMC4415918; doi:10.1371/journal.pone.0125491)

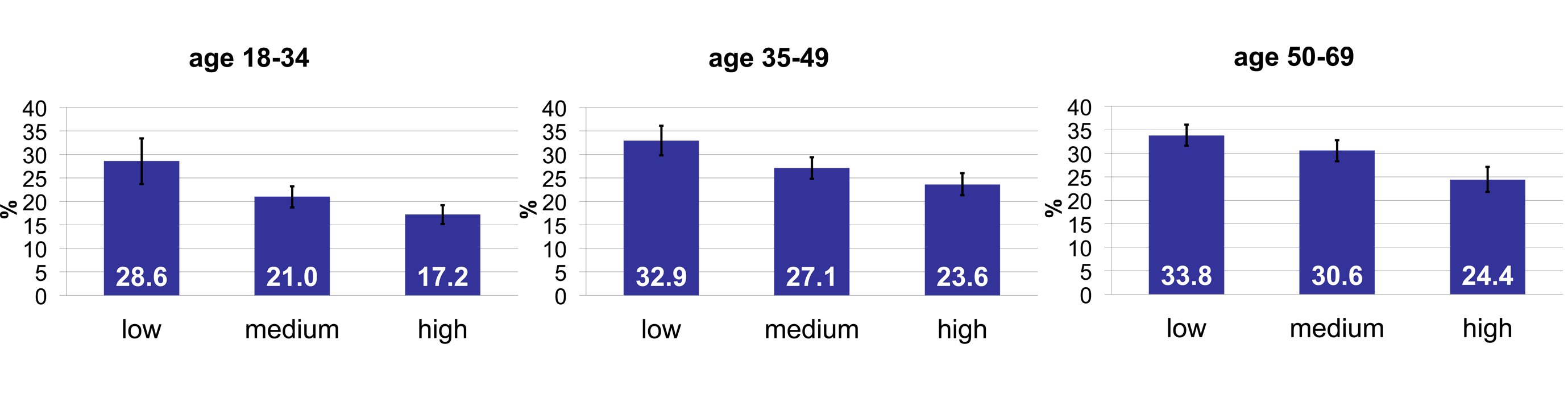

Supplement: S1 Fig — Italian Behavioural Risk Factor Surveillance System’s data (PASSI Toscana, 2009–2012) [33]. Overall, among people aged 18–69 years with an Italian citizenship (n = 13,262) the prevalence of a sedentary lifestyle is 26.5% (95% CI 25.7% to 27.4%). (TIF) [file pone.0125491.s002.tif]

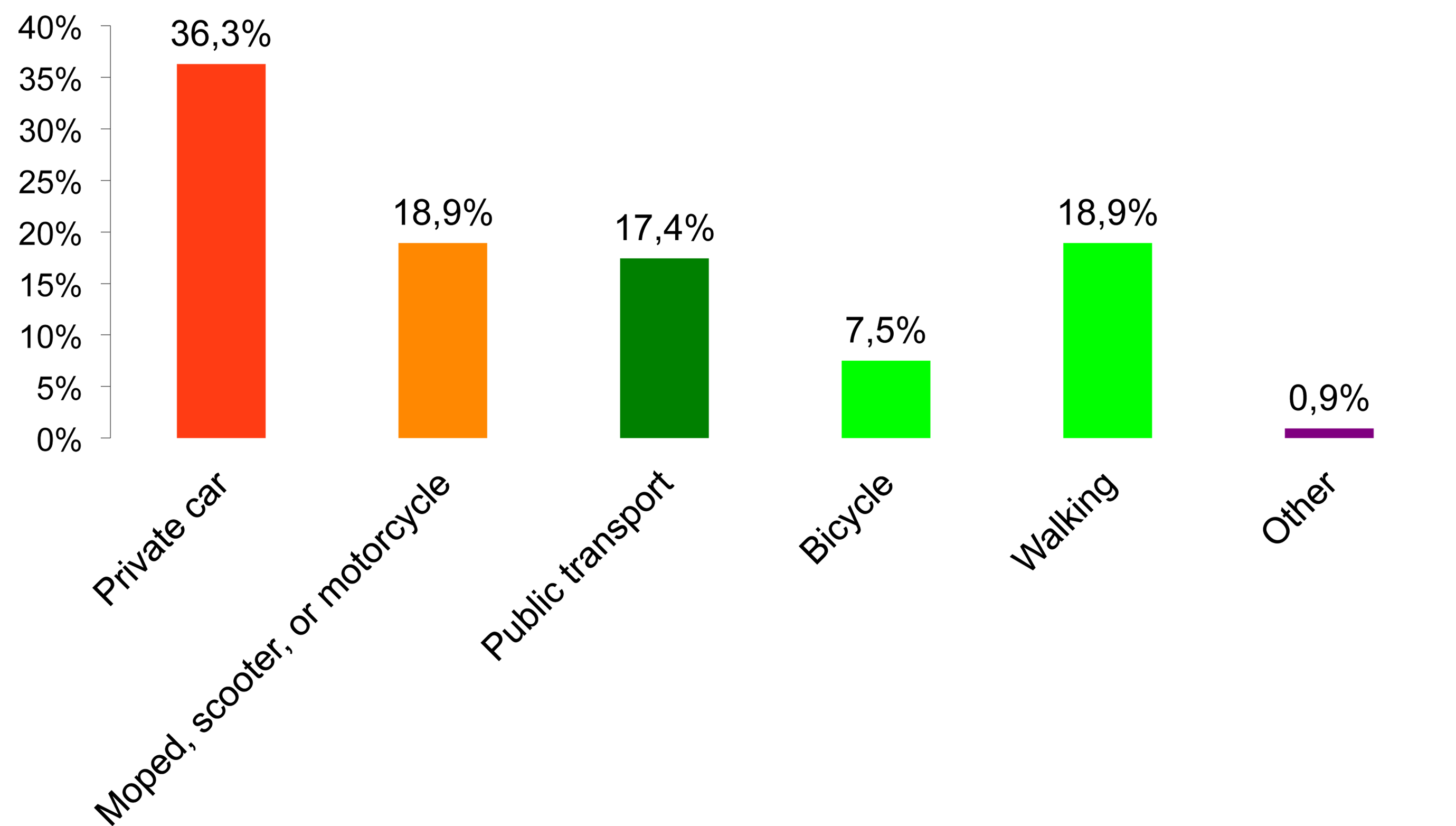

Supplement: S2 Fig — Italian National Institute of Statistics (ISTAT), 2011 population census [34]. (TIF) [file pone.0125491.s003.tif]
