# Supplementary material for: Cycling Promotion and Non-Communicable Disease Prevention: Health Impact Assessment and Economic Evaluation of Cycling to Work or School in Florence
Source: PLoS One. 2015 Apr 30;10(4):e0125491. doi: 10.1371/journal.pone.0125491 (PMC4415918; doi:10.1371/journal.pone.0125491)
Supplement: S1 Table — Italian National Institute of Statistics (ISTAT), 2011 population census [34]. (DOCX) [file pone.0125491.s007.docx]

**S1 Table. Florence population by sex and age-group, 2011 population census data.** Italian National Institute of Statistics (ISTAT), 2011 population census [34].

|  | **Students** | | **Workers** | | **Residents** | |
| --- | --- | --- | --- | --- | --- | --- |
| **Age** | **Males** | **Females** | **Males** | **Females** | **Males** | **Females** |
| 15-19 | 5,915 | 5,778 | 417 | 276 | 6,843 | 6,547 |
| 20-24 | 3,454 | 4,113 | 2,961 | 2,278 | 7,390 | 7,287 |
| 25-29 | 1,889 | 2,131 | 5,714 | 5,467 | 8,266 | 8,768 |
| 30-34 | 835 | 955 | 8,537 | 8,185 | 10,201 | 10,831 |
| 35-39 | 480 | 580 | 11,145 | 10,878 | 12,711 | 13,708 |
| 40-44 | 301 | 455 | 12,395 | 11,890 | 14,006 | 14,970 |
| 45-49 | 259 | 299 | 12,515 | 11,843 | 14,061 | 14,978 |
| 50-54 | 152 | 231 | 10,205 | 10,103 | 11,598 | 13,182 |
| 55-59 | 105 | 119 | 8,185 | 7,870 | 10,489 | 12,068 |
| 60-64 | 51 | 44 | 4,499 | 2,935 | 10,688 | 12,729 |
| 65+ | 123 | 69 | 4,126 | 1,681 | 37,615 | 56,465 |
| **Total** | **13,564** | **14,774** | **80,699** | **73,406** | **143,868** | **171,533** |
